# A statistical modelling approach for determining the cause of reported respiratory syndromes from internet-based participatory surveillance when influenza virus and SARS-CoV-2 are co-circulating

**DOI:** 10.1371/journal.pdig.0000655

**Published:** 2024-12-09

**Authors:** Scott A. McDonald, Albert Jan van Hoek, Daniela Paolotti, Mariette Hooiveld, Adam Meijer, Marit de Lange, Infectieradar team, Arianne van Gageldonk-Lafeber, Jacco Wallinga

**Affiliations:** 1 Centre for Infectious Disease Control, Netherlands National Institute for Public Health and the Environment, Bilthoven, the Netherlands; 2 Institute for Scientific Interchange Foundation, Torino, Italy; 3 Nivel, Utrecht, the Netherlands; 4 Department of Biomedical Data Sciences, Leiden University Medical Center, Leiden, the Netherlands; King’s College London, UNITED KINGDOM OF GREAT BRITAIN AND NORTHERN IRELAND

## Abstract

Symptom-only case definitions are insufficient to discriminate COVID-like illness from acute respiratory infection (ARI) or influenza-like illness (ILI), due to the overlap in case definitions. Our objective was to develop a statistical method that does not rely on case definitions to determine the contribution of influenza virus and SARS-CoV-2 to the ARI burden during periods when both viruses are circulating. Data sources used for testing the approach were weekly ARI syndrome reports from the Infectieradar participatory syndromic surveillance system during the analysis period (the first 25 weeks of 2022, in which SARS-CoV-2 and influenza virus co-circulated in the Netherlands) and data from virologically tested ARI (including ILI) patients who consulted a general practitioner in the same period. Estimation of the proportions of ARI attributable to influenza virus, SARS-CoV-2, or another cause was framed as an inference problem, through which all data sources are combined within a Bayesian framework to infer the weekly numbers of ARI reports attributable to each cause. Posterior distributions for the attribution proportions were obtained using Markov Chain Monte-Carlo methods. Application of the approach to the example data sources indicated that, of the total ARI reports (total of 11,312; weekly mean of 452) during the analysis period, the model attributed 35.4% (95% CrI: 29.2–40.0%) and 27.0% (95% CrI: 19.3–35.2%) to influenza virus and SARS-CoV-2, respectively. The proposed statistical model allows the attribution of respiratory syndrome reports from participatory surveillance to either influenza virus or SARS-CoV-2 infection in periods when both viruses are circulating, but comparability of the participatory surveillance and virologically tested populations is important. Portability for use by other countries with established participatory respiratory surveillance systems is an asset.

## Author summary

Internet-based participatory surveillance systems are valuable public health tools for monitoring illness due to infectious diseases, but when two respiratory viruses are circulating simultaneously, but lead to similar symptoms, case definitions cannot be used to distinguish the virus causing the illness. Our goal was to develop a statistical method that does not rely on case definitions to determine the contribution of influenza virus and SARS-CoV-2 to reported cases of acute respiratory infection. We tested the method using data collected from the Infectieradar system during the 2021/2022 influenza season in the Netherlands, in a period in which the omicron variant of SARS-CoV-2 was also circulating. Our study indicates that the model is able to attribute illness to the possible causes, and is of potential value for other countries with established participatory respiratory surveillance systems.

## Introduction

Internet-based participatory syndromic surveillance systems have proven value for providing (near) real-time information on the spread of seasonal influenza in the general population [1,2,3], through voluntary self-reporting of respiratory symptoms that are consistent with influenza-like illness (ILI). Trends in self-reported ILI have been demonstrated to correlate well with trends in influenza virus infection as determined using other surveillance systems [1,2,4].

As COVID-19 appears to be on the path towards becoming an established recurring infectious disease, temporal overlap with seasonal influenza virus is inevitable. To maximise their utility for tracking either infection, syndromic surveillance systems will ideally need to distinguish syndrome reports attributable to the two (or more) causal agents, especially as several countries intend these systems to serve in the future as a monitor of the community spread of SARS-CoV-2 infection in the absence of widespread testing and collection of incident case data. For instance, in the Netherlands as of 11 April 2022, undergoing a confirmation PCR test at a governmental facility was no longer advised following a positive self-test (rapid antigen test).

Symptom-only case definitions are insufficient to discriminate between ILI and COVID-like illness, due to the overlap in symptoms comprising the two case definitions (S2 Fig). This issue is apparent for the Netherlands in 2022, where the influenza season [5] partially overlapped with a period in which the SARS-CoV-2 positivity rate from national surveillance also exhibited a peak [6], but with different temporal dynamics.

The main aim of this work is to propose a method using Bayesian statistical modelling to perform syndrome attribution, which we test by combining data from the Infectieradar participatory surveillance system in the Netherlands with data from virological testing of GP patients with acute respiratory infection (ARI). This is an inference problem, the goal of which is the estimation of the weekly numbers of ARI reports that are attributable to each cause. Carrying out this inference within a Bayesian framework provides the flexibility to integrate additional relevant data, such as the prevalence of symptom(s) highly specific for one of the causes. Like broadly similar statistical approaches developed to attribute mortality to influenza virus and other respiratory pathogens [7,8], ecological assumptions are required when the data indicating confirmed influenza virus and SARS-CoV-2 infection are not collected from the same participants who self-report respiratory symptom(s). The ultimate goal of developing this methodology is two-fold: first, we wish to better estimate the incidence of both infections (influenza virus and SARS-CoV-2) among persons with ARI in the community (not only among people who consult their GP). Second, to support this effort, we would like to establish an operational approach to provide a clear estimate of the number of virological tests that are required to attribute respiratory syndrome occurrence to the causal agent. The latter is especially important for assessing the resources needed in terms of laboratory testing to implement a cost-effective approach for syndrome attribution, while at the same time leveraging the scale-up capabilities of participatory surveillance.

## Methods

We consider the task of estimating the proportions of respiratory syndromes that are due to influenza virus, SARS-CoV-2, or another pathogen/cause, as an attribution problem, which is solved within a Bayesian framework by essentially inferring the relationships between variation in influenza positives and SARS-CoV-2 positives over time from virological testing, and variation in the time-series of ARI syndrome reports from participatory surveillance. The analysis period was defined as ISO weeks 1–25 of 2022, and was chosen to encompass the 2021/2022 influenza season, which lasted from week 8 through week 20 [5].

### Data sources and case definitions

The method requires two main data sources: syndromic reports from participatory surveillance systems and a second source consisting of virological testing of swabbed patients for both influenza virus and SARS-CoV-2. Ideally the characteristics (e.g., demographic variables, symptom severity) of the tested population should match the characteristics of the participants self-reporting the syndrome of interest. This is not essential for presenting and developing the methodology, but will be important in practice. Virological testing of hospitalised patients could also serve as a viable data source, but then interpretation of the resulting attributions must take into account differences in the severity of illness and testing strategies.

### Syndromic surveillance

The Infectieradar web-based syndromic surveillance system (participatory surveillance) was set up in mid-March 2020 and collates data on symptom occurrence, SARS-CoV-2 testing, vaccination against influenza virus and COVID-19, absenteeism, etc. through weekly surveys filled out by the respondents. As of June 2022, there were approximately 9000 active participants. From Infectieradar data, we calculated the number of submitted surveys per week that matched the case definition used by the European Centre for Disease Control [9] for acute respiratory infection. ARI is defined as at least one of the following symptoms: sore throat, cough, shortness of breath, rhinitis, with sudden onset of symptom(s) indicated [10]. ARI was chosen as syndrome case definition to match the study population of the virological testing data (described below). As in previous studies of involving Infectieradar and other national participatory surveillance systems belonging to the InfluenzaNet monitoring network [11], exclusion criteria were applied to reduce the chance of selection bias (i.e., persons who only registered and/or completed a single survey, likely because of prevalent symptoms) [4,12]. Namely, weekly surveys for which symptom onset date was prior to or on the date of registration were removed, and a participant’s first-ever weekly survey was excluded. Note that a given participant can contribute data to multiple weeks of the analysis period if symptoms persist or reappear; thus we model *prevalent* rather than *incident* syndrome occurrence.

Loss of smell or taste has a high specificity for SARS-CoV-2 infection [13] but occurs infrequently in influenza cases [14]. We retrieved the weekly prevalence of this ’joint’ symptom among Infectieradar ARI reports, to improve the positive predictive value of ARI for SARS-CoV-2 (see below).

### Virological testing

The laboratory testing study population we used to develop and test the attribution model consisted of persons with ARI (consisting of primarily ILI patients, but also non-ILI ARI patients were swabbed) consulting a general practitioner (GP) belonging to a long-established sentinel network of GP practices (the Nivel Primary Care Database; [15]). ARI patients were judged as such by the GP, and were neither systematically nor randomly sampled. GPs were requested to sample at least two ARI patients per week, of which at least one with ILI and one younger than 10 years old. If no ILI patients presented, then ARI patients were sampled. Also, if patients had tested SARS-CoV-2 positive at home using a rapid antigen test they would normally not be coded as ARI but with a specific ICPC code for COVID-19 cases. Such patients would thus be excluded from sampling for virological testing. We note that this sampling protocol is adequate for purposes of developing the attribution method, but not for accurate attribution. Swabs (nose and throat swab) were tested for influenza A virus (with subtyping for A(H1N1)pdm09 and A(H3N2)), influenza B virus (with lineage determination for B/Victoria and B/Yamagata), and SARS-CoV-2 at RIVM’s Centre for Infectious Disease Research, Diagnostics and Laboratory Surveillance (IDS) (for further details on the virological testing procedure, see [16]). Samples are additionally tested for RSV, rhinovirus, enterovirus, other human coronaviruses (hCoV-229E, -OC43, -HKU1, -NL63), parainfluenza viruses (type 1, 2, 3, 4), and human metapneumovirus. Over our 25-week analysis period a total of 1074 samples were tested (mean of 43 samples per week; range 6 to 87).

The 2021/22 influenza season in the Netherlands began later than in a typical season, lasting from week 8 through week 20, with mainly influenza virus type A being detected, of which A(H3N2)

was dominant, with lower proportions of A(H1N1)pdm09 and influenza virus type B/Victoria lineage detected [5]. The peak in ILI incidence was reached approximately in week 15. The pattern for SARS-CoV-2 differed; from national surveillance of the weekly percent positive among tested persons, an earlier peak in week 10 (6.0% SARS-CoV-2 positive) was apparent [6],

### Attribution model

The attribution model consisted of the following set of equations, in which index *i* to refers to week number of the analysis period, and the index *c* to refer to the (unknown) cause of the syndrome report.

We model the observed data, *Z*_i_, which is a summation of the (unknown) number of ARI syndrome reports per cause (i.e., *Y*_influenza,i_ + *Y*_SARS-CoV-2,i_ + *Y*_other,i_) using the *sum* sampler in JAGS [17]:

$$Z_{i}=\sum_{c}Y_{c,i}$$ (1)

The weekly number of syndrome reports attributable to three ’cause categories’: influenza virus, SARS-CoV-2, and other (which, besides other respiratory pathogens, may include conditions such as allergies that can give rise to ARI symptoms), were each assumed to follow a Poisson distribution (appropriate for modelling event counts within a given time interval). *Y*_s,a,i_ is the number of ARI syndrome reports in week *i* attributable to cause *c*.


$$Y_{c,i}∼Poisson(\lambda_{c,i})$$ (2)

The Poisson mean parameter, *λ*_*c*,*i*_, is a function of the probability that the occurrence of the syndrome is attributable to cause *c*, *π*_*c*,*i*_, and a scaling factor, δ_*c*_, which can be interpreted as the expected number of ARI reports per unit probability of an ARI case being attributable to cause *c*. (Reliable estimation of δ_*c*_ requires only that the proportions positive for each cause category from virological testing do not closely co-vary, and that there is variation over time.)

$$\lambda_{c,i}=\delta_{c}\pi_{c,i}$$ (3)

Virological testing data (weekly proportion of samples positive for cause *c*, where *n* = number of positives; *N* = number of tested samples) are assumed binomially distributed (i.e., describing the number of outcomes out of *N* trials); these data inform the probability parameter *p*_*c*,*i*_, for which a non-informative Jeffreys prior, Beta(0.5,0.5), is specified:

$$n_{c,i}∼Binomial(N_{c,i}{,p}_{c,i})$$ (4)

A vague half-normal prior is specified for the scaling factor δ_*c*_ (to ensure a positive density over the non-negative real numbers), with large variance:

$$\delta_{c}∼half-Normal(0,100000)$$ (5)

Next, we bring in further information on the weekly prevalence (*prev*_*i*_) of highly-specific symptom *loss of smell/taste*, updating *p*_*SARS*−*CoV*−2,*i*_, the prior probability of SARS-CoV-2 given ARI as determined from virological testing (*w* = number of ARI reports with symptom; *W* = total number of ARI reports), using Bayes’ theorem (e.g., [18]). This requires estimates of the sensitivity (*sens*) and specificity (*spec*) of the symptom *loss of smell/taste* for SARS-CoV-2 infection; these parameters (0.487 and 0.954, respectively) were taken from a published analysis of UK first wave data collected using the COVID Symptom Study app [13].


$$w_{i}∼Binomial(W_{i}{,prev}_{i})$$ (6A)

$${\hat{p}}_{c,i}=\frac{\left(sens*p_{c,i}\right)}{\left(sens*p_{c,i})+(1-spec)(1-p_{c,i}\right)}$$ (6B)

Because this updated prior applies only to individuals who reported loss of smell/taste in week *i*, we define the probability that the ARI report was due to SARS-CoV-2 infection, *π*_*SARS*−*CoV*−2,*i*_, as a weighted function of the prevalence of loss of smell/taste in that week, and we set *π*_*influenza*,*i*_ equal to *p*_*influenza*,*i*_.


$$\pi_{c,i}=(1-{prev}_{i})p_{c,i}+{prev}_{i}({\hat{p}}_{c,i})$$ (7)

### Implementation

After discarding the first 10,000 iterations as burn-in, 3000 samples from the posterior distribution were taken to allow inference of the model parameters. Three chains were run, and results examined for adequate mixing and convergence (see Supporting Information, S4 Fig for posterior densities for key parameters). We report (and plot) posterior median estimates and define 95% credible intervals (CrIs) using the 2.5% and 97.5% quantiles of the posterior distribution. Sampling was carried out within JAGS [17], accessed via the runjags package [19] for R [20]. JAGS code is provided in the Supporting Information, S1 Code.

To prevent information flow from the observed data on weekly ARI syndrome reports to the posterior probabilities that the ARI report was due to influenza virus or to SARS-CoV-2 infection (i.e., the posterior distributions for the parameters *π*_*influenza*,*i*_ and *π*_*SARS*−*CoV*−2,*i*_ should reflect the number of positives and number of tests only), virological testing data are introduced in a separate JAGS ’data block’. As only one (random) sample from the parameters *p*_*c*,*i*_ are taken per model run (in the JAGS data block), we aggregated the posterior samples from 200 runs of the model, from which the posterior medians and 95% CrIs were extracted.

Given that the Infectieradar weekly survey also queried whether participants had undergone testing (SARS-CoV-2 PCR/antigen test) since their previous survey, we could compare the modelled proportion of ARI patients attributed to SARS-CoV-2 infection to the observed proportion of Infectieradar participants matching the ARI case definition who in the same week (or in the subsequent week, as they may not have tested immediately upon symptom onset) reported a positive test result.

### Sensitivity analyses

#### Sensitivity analysis 1

For application of the approach to other settings, it is of interest to determine the impact of data availability–namely, as mentioned in the Introduction, the total number of swabbed ARI patients from sentinel surveillance undergoing virological testing–on the attribution estimates. This will allow estimation of the minimum weekly number of tested patients required for the obtaining valid attribution estimates from the model. We conducted a sensitivity analysis by successively reducing the total size of the testing dataset through the range (90% through 10% of the original size, in steps of 20%) by randomly removing data points (e.g., 90% of 1074 = 107 removed, 70% of 1074 = 322 removed, etc), and re-running the model. For each step size, we ran the random removal procedure 20 times, aggregating the posterior samples for the 20 runs, and then calculated the posterior median influenza virus and SARS-CoV-2 attribution estimates and associated 95% credible intervals. We also calculated the root mean-squared error (RMSE), which quantifies the degree of discrepancy to the attribution estimations derived using the full dataset (S1 Table). To give greater weight to larger than smaller discrepancies, we chose RMSE as the loss function.

#### Sensitivity analysis 2

Prior distribution assumptions were assessed for the probability parameters *p*_*c*,*i*_, by substituting the Jeffreys prior, Beta(0.5,0.5) with the uniform Bayes-Laplace prior, Beta(1,1) (sensitivity analysis 2A), and for the scaling factors δ_*c*_, by substituting the vague half-Normal prior with an alternative, Jeffrey’s prior for the Poisson rate parameter (which is proportional to δ_*c*_^-0.5^) (sensitivity analysis 2B), that also ensured a positive density over the non-negative real numbers.

## Results

A total of 11,312 syndrome reports fulfilled the ARI case definition during our 25-week analysis period (per-week mean of 452), out of a total of submitted 229,299 surveys (per-week mean of 9172). The age-group 45–64 years contributed the highest percentage of ARI syndrome reports during weeks 1–25 of 2022 (54%), followed by the 15–44 years age-group (25%).

**Fig 1:**
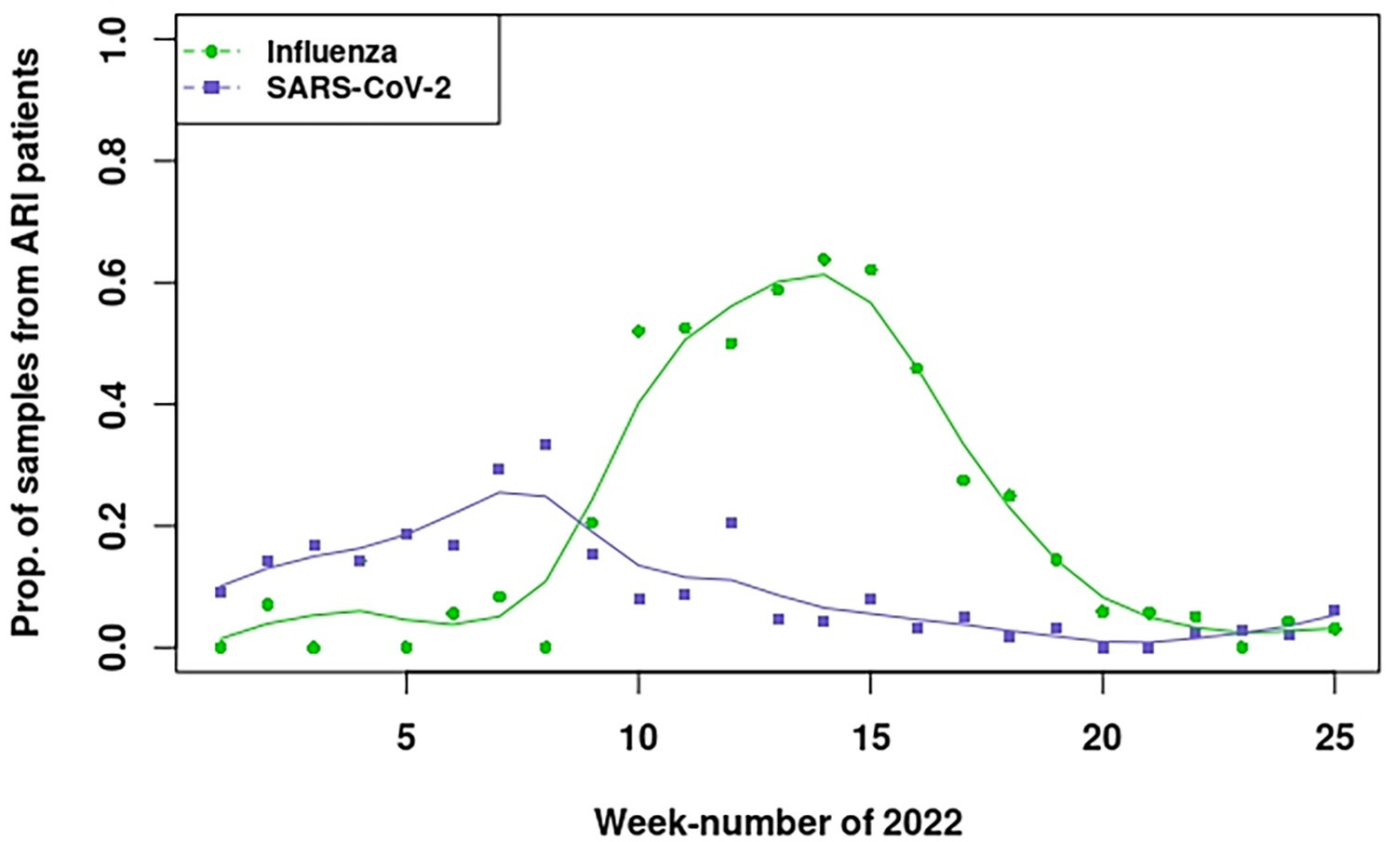
Weekly observed proportions of influenza-positive and SARS-CoV-2 positive results among sampled ARI patients from sentinel GP surveillance (i.e., number of virus-positive results divided by the number of samples tested per week). Cubic smoothing splines (10 df) are superimposed.

The proportions of influenza virus and SARS-CoV-2 positive results from virological testing of ARI patients from the sentinel GP network, with fitted cubic smoothing splines, are shown in Fig 1. In comparison with SARS-CoV-2, influenza activity appeared to rise more steeply, peaking approximately 7–8 weeks later. Over the 25-week analysis period, influenza virus and SARS-CoV-2 were detected in 30.6% and 7.8% of samples, respectively. Other viruses were detected as follows: RSV (4.6%), rhinovirus (13.9%), enterovirus (1.2%), other human coronaviruses (9.7%), parainfluenza viruses (7.5%), and human metapneumovirus (1.9%) (NB. double infections could occur). The highest percentage of ARI patients swabbed between 2021/week 40 and 2022 week 20 was for the age-group 15–44 years (approximately 35%), followed by the 45–64 years age-group (approximately 27%) [5].

**Fig 2:**
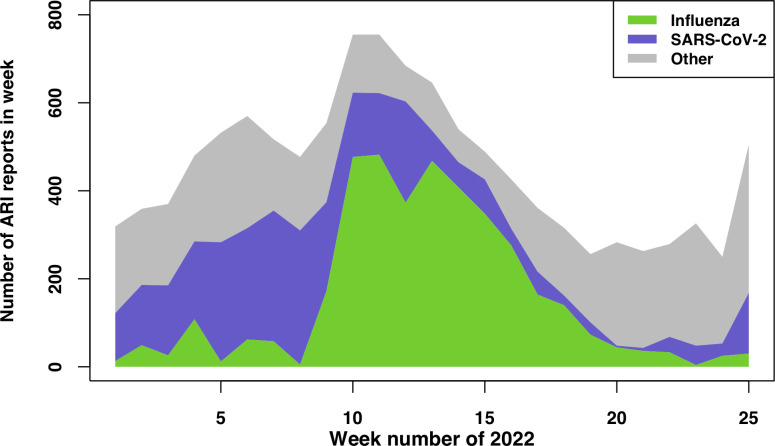
Posterior estimates from the attribution model of the weekly number of ARI syndrome reports per cause, over the 25-week analysis period.

**Fig 3:**
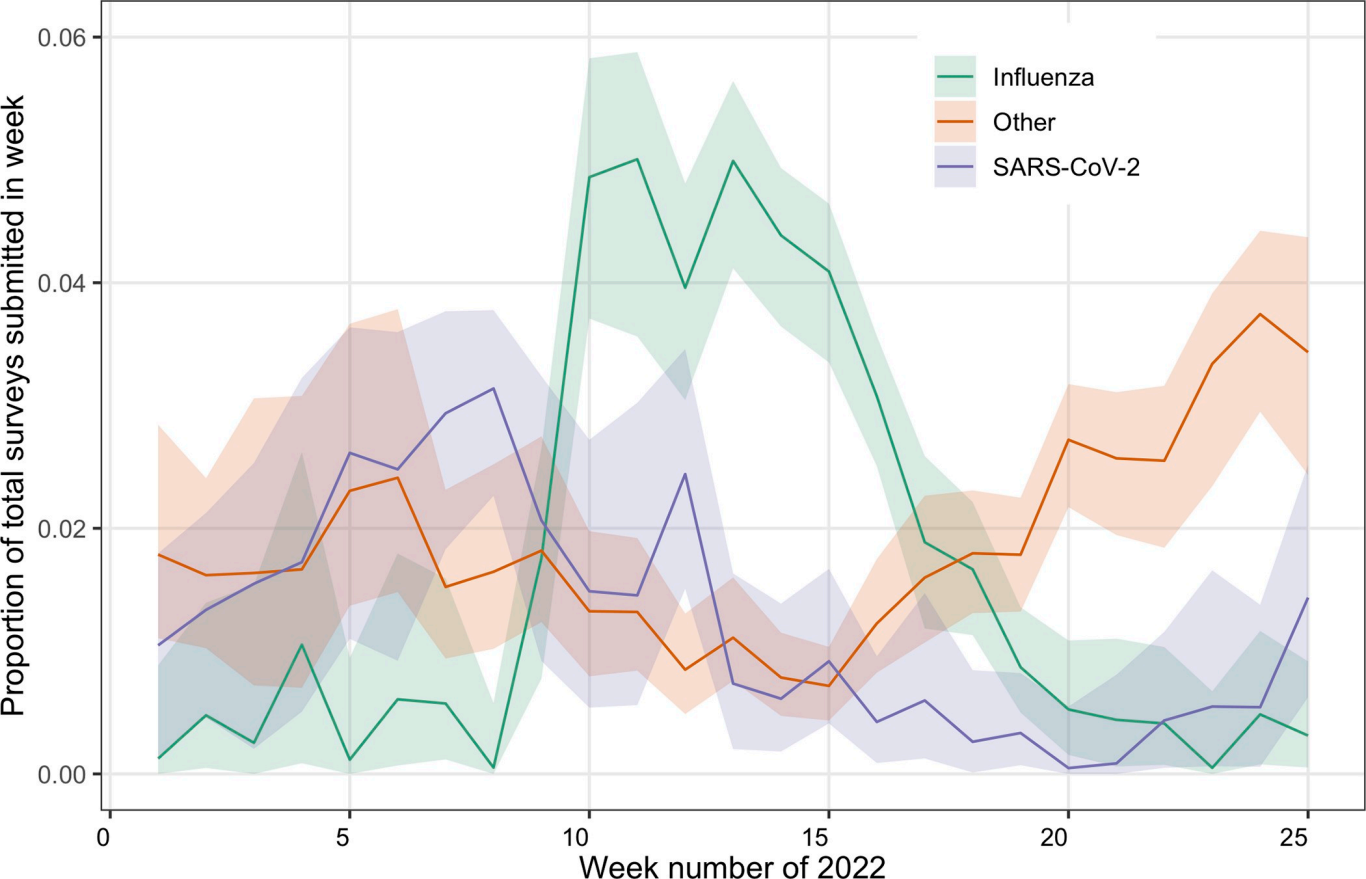
Posterior estimates from the attribution model of the weekly proportions per cause (out of total weekly submitted surveys), over the 25-week analysis period.

The model-estimated number of ARI syndrome reports per week attributable to infection with influenza virus, SARS-CoV-2 infection, or to other causes is depicted in Fig 2. Of the total ARI reports during the defined influenza season (week 8 through week 20), the model attributed 52.7% (95% CrI: 45.1–58.2%) and 21.3% (95% CrI: 14.6–29.9%) to influenza virus and SARS-CoV-2, respectively. Fig 3 shows the estimated proportions of the total surveys submitted in each week that were attributed to influenza virus, to SARS-CoV-2 and to another cause. Over the whole analysis period, 35.4% (95% CrI: 29.2–40.4%) and 27.0% (95% CrI, 19.3–35.2%) of ARI was estimated to be attributable respectively to influenza virus and to SARS-CoV-2 (but we note the limitations–see Methods and Discussion –in interpreting the attributed percentages given the protocol for selecting patients for swabbing, and differences in healthcare-seeking behaviour between persons who tested SARS-CoV-2 positive using a home test and those who did not). Influenza-attributable ARI reached a peak of 5.0% of all submitted surveys (in week 11), whereas SARS-CoV-2 attributable ARI did not exceed 3.1% (in week 8) within our analysis period (Fig 3).

Posterior estimates of the scaling factors, δ_*c*_, were 707 (95% CrI: 579–806), 884 (95% CrI: 603–1269), and 252 (95% CrI: 209–300) per unit of attribution probability for influenza virus, SARS-CoV-2, and other causes, respectively.

Of those Infectieradar participants with ARI who also reported a linked SARS-CoV-2 PCR/antigen test, 26.5% had a positive test outcome. Both model-estimated attribution probabilities and the observed proportion of test-positives are plotted over time in S3 Fig, and indicate broadly consistent temporal trends.

Integrating the prevalence of *loss of smell/taste* in the estimation of the probability of SARS-CoV-2 given ARI made only a small difference to the results (27.0% of total ARI reports over the 25-week analysis period was attributed to SARS-CoV-2 when *loss of smell/taste* was included, compared to 22.6% if excluded), as the prevalence of these highly specific symptoms for SARS-CoV-2 was low throughout the analysis period, reaching their highest levels of 7–8% in week 7 (S2 Fig).

### Sensitivity analyses

S1 Table shows the effect of artificially reducing the size of the virological dataset. The uncertainty in the influenza and SARS-CoV-2 attribution estimates for the defined 13-week influenza season tended to increase with decreasing size of the testing dataset. As well, the degree to which attribution proportions differed from those estimated using 100% of the data, as quantified by RMSE, tended to increase as dataset size decreased. Between 50% and 30% of the original size, there was a relatively large increment in RMSE for influenza virus, with a large increment in RMSE at between 70% and 50% for SARS-CoV-2, which suggests that 70% of our full dataset (i.e., mean of 30 tested ARI patients per week) is a practical minimum for attributing respiratory syndrome to influenza or SARS-CoV-2 (S1 Table).

There was minimal influence from the choice of noninformative prior for the proportions of influenza or SARS-CoV-2 positive lab test results, and the alternative prior choice for the scaling factors δ_*c*_ also did not substantively alter the attribution estimates (S3 Table).

## Discussion

We have presented a statistical modelling approach for the attribution of syndrome self-reports from participatory surveillance to their cause during periods when influenza virus and SARS-CoV-2 are co-circulating, which cannot be adequately distinguished using syndrome-only case definitions. We tested the attribution method using data from Infectieradar and from virological testing of ARI patients from sentinel GP surveillance. Conditional on limitations related to the criteria and the protocol for swabbing ARI patients, the model suggested that a much higher proportion of ARI reports were estimated to be attributed to influenza (53%) than to SARS-CoV-2 (21%) over the 13-week 2022 influenza season in the Netherlands.

Influenza virus activity peaked around week 14–15 (based on the GP sentinel surveillance ILI incidence rate and the test-positive proportion [5]; see Fig 1 in this reference), and there were two COVID-19 peaks that occurred earlier than the influenza peak (according to the weekly number of confirmed SARS-CoV-2 tests at public health service test locations): in week 5 and a smaller peak in week 10 [5]. The time-series of Infectieradar ARI syndrome reports over weeks 1–25 of 2022 therefore reflect a mixture of these two circulating viruses, as well as a sizeable contribution from other (unknown) causal agents (Fig 2). Among those Infectieradar participants whose symptoms matched the ARI case definition who also reported being tested, 26.5% were SARS-CoV-2 positive. This is identical to our overall SARS-CoV-2 attributed proportion (27.0%), and the temporal patterns were roughly consistent (S3 Fig). These similarities, however, should not be interpreted as validation of the model-based attribution estimates given the small proportion of Infectieradar participants who self-selected for, and reported, testing, and the constraints on the sampling of sentinel GP patients and healthcare-seeking behaviour already mentioned (see Methods).

Differences in the estimated scaling factors for influenza virus and SARS-CoV-2 can be cautiously interpreted as between-pathogen differences in the chance that ARI symptoms develop following infection with the particular respiratory pathogen. However, this interpretation must be considered in the context of other possible–but not tested for–causes of the symptoms. Given the ecological design and the constraints on swabbing ARI patients in our data, one cannot definitively say on the basis of a larger scaling factor estimated for influenza that influenza virus infection leads to a higher chance of ARI symptoms compared with SARS-CoV-2.

Advantages of the present approach, which is similar to ecological regression methods employed for attribution of respiratory-associated mortality or hospital admissions over multiple seasons [3,7,21], is that it is relatively simple to implement and requires only virological testing data from a comparable population. Sensitivity analysis 1 has suggested a lower limit for the dataset size (i.e., a mean of 30 ARI patients tested per week) needed to attribute respiratory syndrome occurrence to the causal agent. Unlike additive regression approaches, data from multiple seasons are not required for robust estimates. Furthermore, the Bayesian modelling framework propagates uncertainty from all data sources to the attribution estimates, the prevalence of distinguishing symptoms is straightforward to incorporate, and the approach allows further evidence to be integrated if available; for instance, a second source of weekly SARS-CoV-2 testing data could easily be included.

The main limitation is the ecological approach to estimation, and the consequent key assumption that the population sampled for virological testing is comparable–in susceptibility to infection, symptom severity, and in demographic characteristics such as age–to the respondents reporting the syndrome of interest. Implicit in the use of sentinel GP data is the assumption that health-care seeking behaviour is not conditional on the cause; for instance, if one strongly suspects SARS-CoV-2 infection and goes to a government COVID-19 testing facility rather than to a GP, then the percentage of ARI attributed to SARS-CoV-2 will be under-estimated. Because patients were advised to do a rapid antigen test before going to the GP, and persons testing positive were neither recorded as ARI patients nor swabbed, our virological testing dataset is biased against detecting SARS-CoV-2. Future work will address the validity of the attribution model, as in the following (2022/2023) season a random sample of Infectieradar participants were mailed a rapid antigen test which they were asked to use if they had respiratory symptoms, and–if the result was SARS-CoV-2 positive–to send in the swab to IDS for testing for other viruses including as influenza. This will provide data on SARS-CoV-2 and influenza infection within a single study population.

The tested population from the sentinel GP network consisted of a comparable number of ILI patients and non-ILI ARI patients [5], which may not provide an ideal match to the Infectieradar ARI reports. In addition, because the absolute numbers of syndrome reports attributed to each cause will vary depending on the syndrome case definition (e.g., greater weekly numbers of influenza- and SARS-CoV-2 attributed syndrome reports would be estimated when based on COVID-19-like illness instead of ARI, or on the more specific ILI case definition; S1 Fig), the proportion of syndrome reports attributed to each cause is a more appropriate outcome measure. We have made some simplifications for the purpose of illustrating the method. Namely, the sensitivity and specificity of loss of smell/taste are likely variant-specific; using the first wave (ancestral variant) estimates for the current omicron-era data will introduce some inaccuracies. Finally, the time-series of both ARI syndrome reports and virological testing are assumed to align; however, the onset of ARI (in both Infectieradar and sentinel GP patients) may occur a few days before being tested.

In summary, the proposed method can assist in attributing respiratory syndrome reports from participatory surveillance to either influenza virus or SARS-CoV-2 infection in periods when both viruses are circulating. The approach is particularly interesting because it can be easily exported to other countries with implemented participatory surveillance systems. The estimated lower bound in the weekly number of swabs submitted for virological testing provided by sensitivity analysis 1 supports the feasibility of the present approach in other countries where participatory surveillance data are available but routine virological testing is less well-established than in the Netherlands.

## Supporting information

S1 Table Result of sensitivity analysis 1, in which the size of the virological testing dataset is varied. (DOCX)

S2 Table Data from Infectieradar participatory surveillance. (DOCX)

S3 Table Result of sensitivity analysis 2, in which alternative prior distributions for two parameters are tested. (DOCX)

S1 Fig Weekly prevalence of three respiratory syndromes in weeks 1–25 of 2022. (DOCX)

S2 Fig Prevalence of symptoms highly specific for SARS-CoV-2 infection. (DOCX)

S3 Fig Comparison of the proportion of weekly Infectieradar ARI reports attributed to SARS-CoV-2 with the proportion of weekly ARI reports with a linked positive test result. (DOCX)

S4 Fig Trace plots and posterior densities for seven key model parameters. (DOCX)

S1 Code JAGS code. (DOCX)
