# Supplementary material for: A statistical modelling approach for determining the cause of reported respiratory syndromes from internet-based participatory surveillance when influenza virus and SARS-CoV-2 are co-circulating
Source: PLOS Digit Health. 2024 Dec 9;3(12):e0000655. doi: 10.1371/journal.pdig.0000655 (PMC11627408; doi:10.1371/journal.pdig.0000655)
Supplement: S1 Table — (DOCX) [file pdig.0000655.s001.docx]

**S1 Table.** Result of sensitivity analysis to the size of the virological testing dataset, evaluated through comparison of attribution estimates and root mean-squared error (RMSE). In the rows for '90%' and below, posterior median attribution proportions over the 13-week influenza season (with weekly number of ARI reports as denominator) and associated 95% credible interval (CrI) represent aggregation over 50 runs (in which the dataset was reduced by random removal of data-points) are shown.

**Number of samples Influenza attribution SARS-CoV-2 attribution**

***n* (% of full dataset) Median (95% CrI) RMSE Median (95% CrI) RMSE**

*1074 (100%) 52.7% (44.1-58.2%) -- 21.3% (14.6-29.9%) --*

967 (90%) 52.3% (45.0-57.9%) 5.9 20.1% (14.8-28.6%) 8.2

752 (70%) 51.9% (41.6-59.6%) 9.3 19.9% (9.5-32.2%) 19.6

537 (50%) 53.3% (40.9-61.5%) 11.9 15.6% (7.3-31.3%) 39.1

322 (30%) 52.9% (39.9-64.0%) 22.2 13.1% (5.2-31.6%) 60.3

107 (10%) 49.3% (23.4-67.0%) 46.9 13.4% (3.3-28.0%) 83.6
