# Supplementary material for: A statistical modelling approach for determining the cause of reported respiratory syndromes from internet-based participatory surveillance when influenza virus and SARS-CoV-2 are co-circulating
Source: PLOS Digit Health. 2024 Dec 9;3(12):e0000655. doi: 10.1371/journal.pdig.0000655 (PMC11627408; doi:10.1371/journal.pdig.0000655)
Supplement: S2 Table — (DOCX) [file pdig.0000655.s002.docx]

**S2 Table.** Data from Infectieradar participatory surveillance and from virological testing of GP patients (from Diagnostics and Laboratory Surveillance, Centre for Infectious Disease Research, RIVM) used to test the attribution model. These are: the total number of Infectieradar participants per ISO weeknumber of 2022 reporting symptoms conforming to the acute respiratory infection (ARI) case definition (*n*_ARI_), the total number of ARI reports in which *loss of smell* and/or *loss of taste* was also reported (*n*_loss_smell_or_taste_), the total number of GP patient samples that were tested (*n*_tested_), and the total number positive for influenza virus (*n*_infl_), the total number positive for SARS_CoV-2 (*n*_SARS-CoV-2_), and the remainder (*n*_other_).

| **Weeknumber** | ***n_ARI_*** | ***n*_loss_smell_or_taste_** | ***n*_tested_** | ***n*_infl_** | ***n*_SARS-CoV-2_** | ***n*_other_** |
| --- | --- | --- | --- | --- | --- | --- |
| 1 | 319 | 27 | 11 | 0 | 1 | 10 |
| 2 | 359 | 21 | 14 | 1 | 2 | 11 |
| 3 | 370 | 18 | 6 | 0 | 1 | 5 |
| 4 | 480 | 24 | 7 | 1 | 1 | 5 |
| 5 | 532 | 35 | 16 | 0 | 3 | 13 |
| 6 | 570 | 36 | 18 | 1 | 3 | 14 |
| 7 | 517 | 51 | 24 | 2 | 7 | 15 |
| 8 | 477 | 30 | 21 | 0 | 7 | 14 |
| 9 | 554 | 42 | 39 | 8 | 6 | 25 |
| 10 | 755 | 67 | 50 | 26 | 4 | 20 |
| 11 | 755 | 46 | 57 | 30 | 5 | 22 |
| 12 | 684 | 46 | 78 | 39 | 16 | 23 |
| 13 | 646 | 44 | 85 | 50 | 4 | 31 |
| 14 | 540 | 40 | 69 | 44 | 3 | 22 |
| 15 | 489 | 39 | 87 | 54 | 7 | 26 |
| 16 | 426 | 23 | 61 | 28 | 2 | 31 |
| 17 | 361 | 28 | 40 | 11 | 2 | 27 |
| 18 | 316 | 13 | 56 | 14 | 1 | 41 |
| 19 | 256 | 13 | 62 | 9 | 2 | 51 |
| 20 | 283 | 13 | 51 | 3 | 0 | 48 |
| 21 | 263 | 18 | 35 | 2 | 0 | 33 |
| 22 | 279 | 21 | 39 | 2 | 1 | 36 |
| 23 | 326 | 14 | 36 | 0 | 1 | 35 |
| 24 | 250 | 20 | 47 | 2 | 1 | 44 |
| 25 | 505 | 46 | 65 | 2 | 4 | 59 |
