# Supplementary material for: A statistical modelling approach for determining the cause of reported respiratory syndromes from internet-based participatory surveillance when influenza virus and SARS-CoV-2 are co-circulating
Source: PLOS Digit Health. 2024 Dec 9;3(12):e0000655. doi: 10.1371/journal.pdig.0000655 (PMC11627408; doi:10.1371/journal.pdig.0000655)
Supplement: S3 Table — (DOCX) [file pdig.0000655.s003.docx]

**S3 Table.** Results of sensitivity analysis 2: the estimated posterior proportions of ARI syndrome reports attributed to influenza and SARS-CoV-2, for the defined influenza season (week 8 through week 20) and for the entire 25-week analysis period. In sensitivity analysis 2A the Jeffreys prior, Beta(0.5,0.5), on parameters $p_{c,i}$ is substituted by the uniform (Bayes-Laplace) prior, Beta(1,1); in sensitivity analysis 2B the half-Normal(0,100000) prior on parameters $\partial_{c}$ is substituted by Jeffrey’s prior for the Poisson rate parameter, 1/sqrt(lambda) (in JAGS approximated by Gamma(0.5,0.00001)).

|  | ***13-week influenza season*** | | ***25-week analysis period*** | |
| --- | --- | --- | --- | --- |
| **Analysis** | **Influenza**  **median (95% CrI)** | **SARS-CoV-2 median (95% CrI)** | **Influenza median (95% CrI)** | **SARS-CoV-2 median (95% CrI)** |
| Main analysis | 52.7% (45.1-58.2%) | 21.3% (14.6-29.9%) | 35.4% (29.2-40.0%) | 27.0% (19.3-35.2%) |
| Sensitivity analysis 2A | 54.2% (47.8-60.8%) | 21.2% (13.1-28.7%) | 37.8% (32.7-43.4%) | 27.2% (16.8-35.6%) |
| Sensitivity analysis 2B | 52.7% (45.5-58.5%) | 20.8% (13.6-30.6%) | 35.3% (30.3-40.6%) | 26.2% (17.8-36.4%) |
