# Supplementary material for: A statistical modelling approach for determining the cause of reported respiratory syndromes from internet-based participatory surveillance when influenza virus and SARS-CoV-2 are co-circulating
Source: PLOS Digit Health. 2024 Dec 9;3(12):e0000655. doi: 10.1371/journal.pdig.0000655 (PMC11627408; doi:10.1371/journal.pdig.0000655)

**S1 Fig**. Weekly prevalence of three respiratory syndromes: COVID-19-like illness (defined as at least one of {fever, cough, shortness of breath, loss of smell, loss of taste}; ECDC, 2020); acute respiratory infection (defined as at least one of {sore throat, cough, shortness of breath, rhinitis}, with sudden onset; EU, 2018); and influenza-like illness (defined as sudden onset and at least one of {fever, fatigue, headache, muscle/joint pain} and at least one of {sore throat, cough, shortness of breath, rhinitis}; EU, 2018). Plotted proportions indicate the number of weekly surveys where symptoms match the respiratory syndrome, among all Infectieradar weekly surveys submitted in weeks 1-25 of 2022.


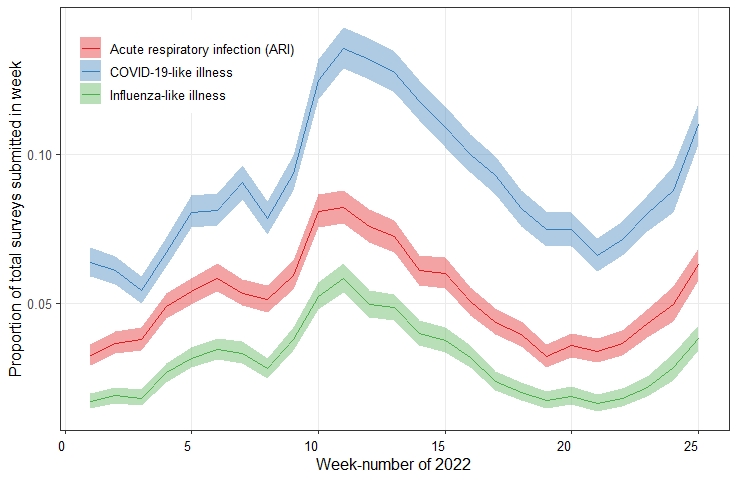

Supplement: S1 Fig — (DOCX) [file pdig.0000655.s004.docx]
