# Supplementary material for: A statistical modelling approach for determining the cause of reported respiratory syndromes from internet-based participatory surveillance when influenza virus and SARS-CoV-2 are co-circulating
Source: PLOS Digit Health. 2024 Dec 9;3(12):e0000655. doi: 10.1371/journal.pdig.0000655 (PMC11627408; doi:10.1371/journal.pdig.0000655)
Supplement: S2 Fig — (DOCX) [file pdig.0000655.s005.docx]

**S2 Fig**. Prevalence of symptoms highly specific for SARS-CoV-2 infection (*loss of smell*, *loss of taste*) among Infectieradar participants who reported symptom(s) matching the ARI case definition. Also shown are two symptoms with high specificity for influenza infection (*headache*: 0.91, *sore throat*: 0.85; Shah et al., 2015) but moderate specificity for SARS-CoV-2 infection (headache: 0.48, sore throat: 0.56; Antonelli et al., 2021).

**
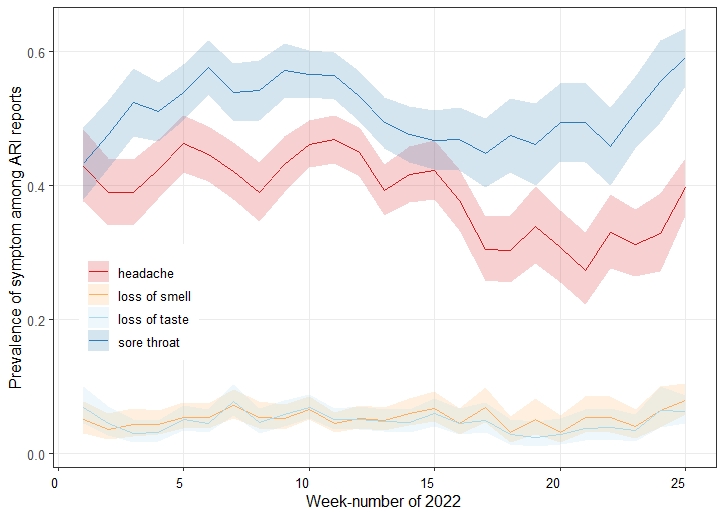
**
