# Supplementary material for: A statistical modelling approach for determining the cause of reported respiratory syndromes from internet-based participatory surveillance when influenza virus and SARS-CoV-2 are co-circulating
Source: PLOS Digit Health. 2024 Dec 9;3(12):e0000655. doi: 10.1371/journal.pdig.0000655 (PMC11627408; doi:10.1371/journal.pdig.0000655)

**S3 Fig.** Comparison of the proportion of weekly Infectieradar ARI reports attributed to SARS-CoV-2 with the proportion of weekly ARI reports with a linked positive test result.


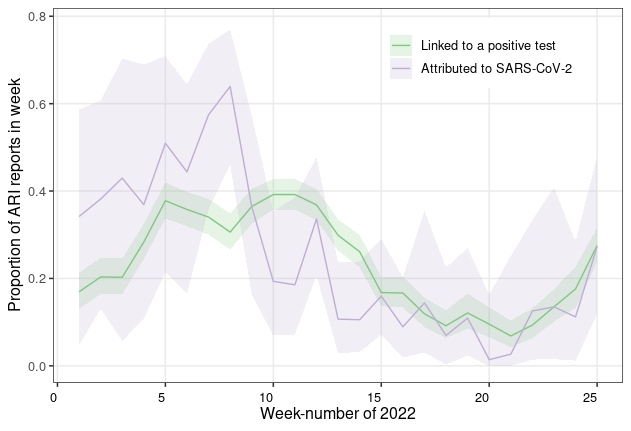

Supplement: S3 Fig — (DOCX) [file pdig.0000655.s006.docx]
