# Supplementary material for: A statistical modelling approach for determining the cause of reported respiratory syndromes from internet-based participatory surveillance when influenza virus and SARS-CoV-2 are co-circulating
Source: PLOS Digit Health. 2024 Dec 9;3(12):e0000655. doi: 10.1371/journal.pdig.0000655 (PMC11627408; doi:10.1371/journal.pdig.0000655)
Supplement: S4 Fig — (DOCX) [file pdig.0000655.s007.docx]

**S4 Fig**. Trace plots and posterior densities for seven key model parameters (derived from 3 chains and *n*=1000 posterior samples per chain, after a burn-in of 10,000 iterations). The x-axis for the left panel of each parameter plot indicates iteration number.

**
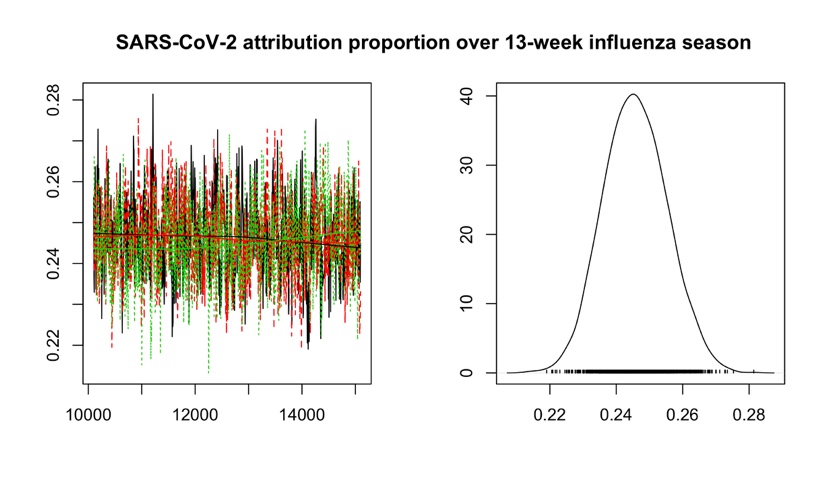

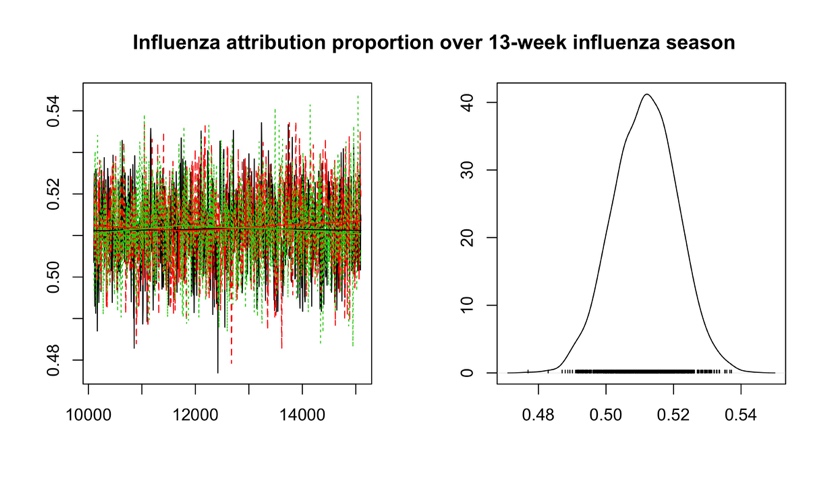
**

**
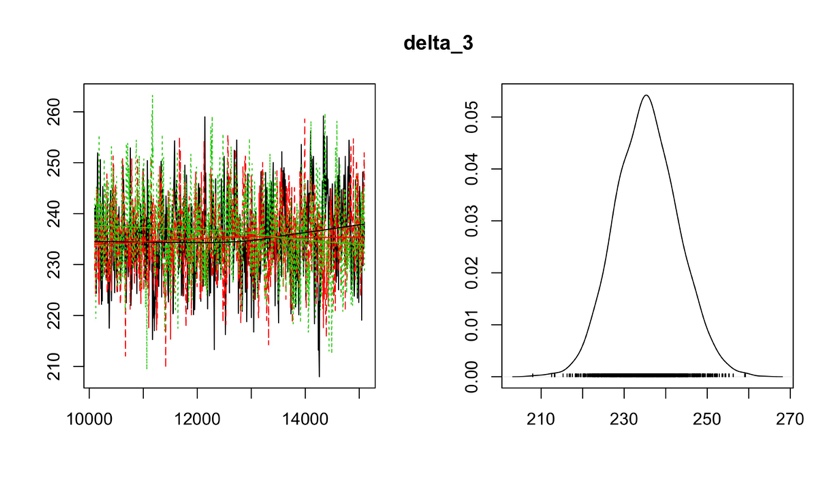

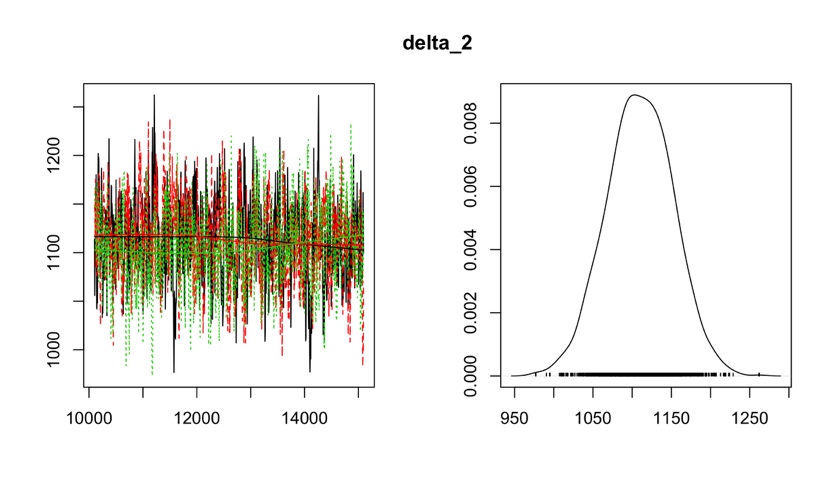
**
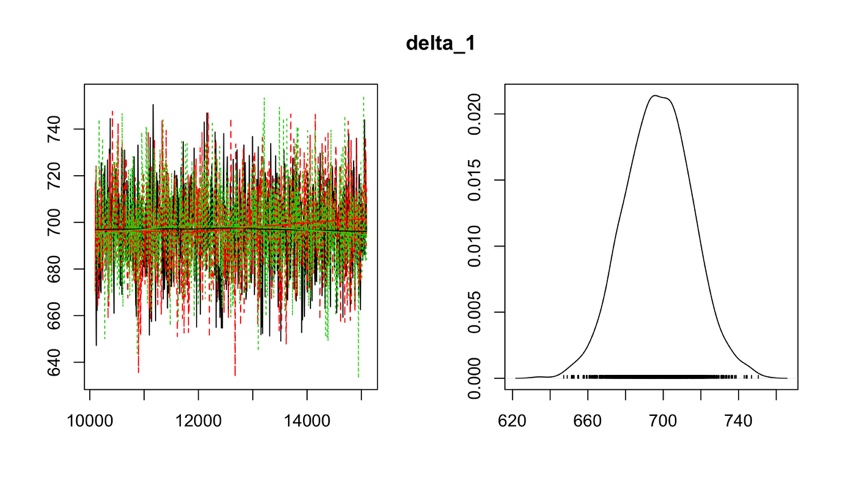
**
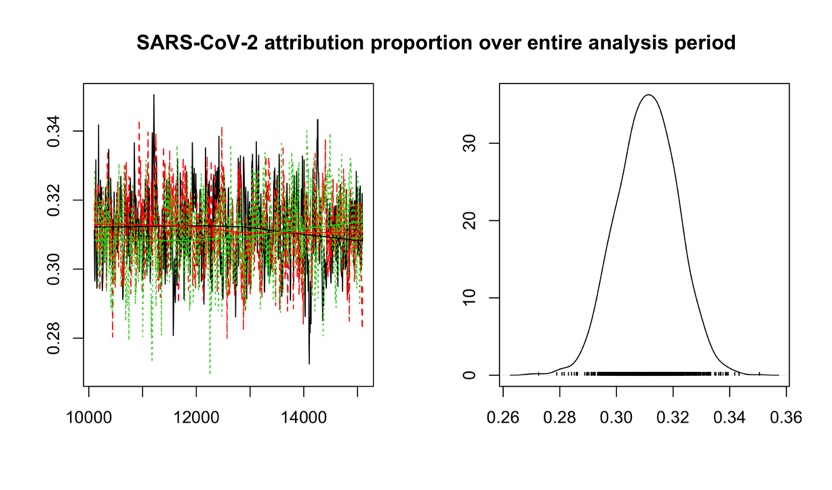

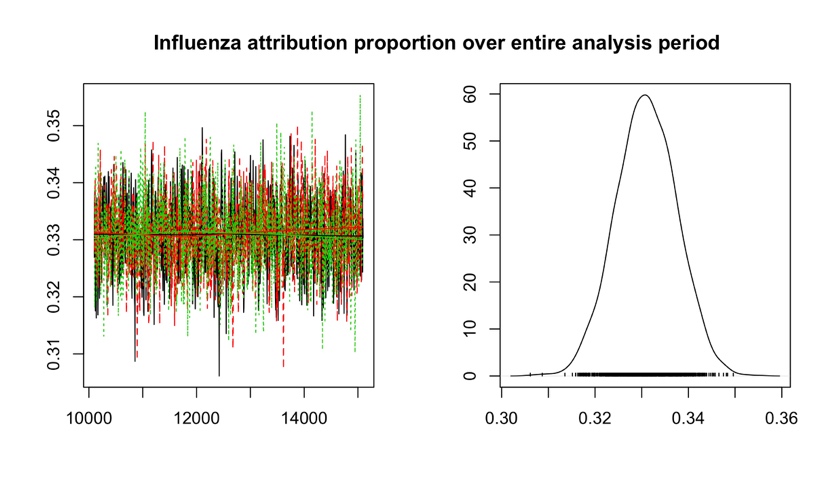
**
