# Supplementary material for: A statistical modelling approach for determining the cause of reported respiratory syndromes from internet-based participatory surveillance when influenza virus and SARS-CoV-2 are co-circulating
Source: PLOS Digit Health. 2024 Dec 9;3(12):e0000655. doi: 10.1371/journal.pdig.0000655 (PMC11627408; doi:10.1371/journal.pdig.0000655)
Supplement: S1 Code — (DOCX) [file pdig.0000655.s008.docx]

**S1 Code**. JAGS code.

data {

for(i in 1:N) { # [loop over N weeks]

# Equivalent to: c1[i] ~ dbin(p.infl.given.syn[i],n1[i]) with Jeffreys prior: dbeta(0.5,0.5):

p.infl.given.syn[i] ~ dbeta(c1[i]+0.5,(n[i]-c1[i]+0.5))

p.sars.given.syn[i] ~ dbeta(c2[i]+0.5,(n[i]-c2[i]+0.5))

p.other.given.syn[i] ~ dbeta(c3[i]+0.5,(n[i]-c3[i]+0.5))

}

}

model {

# Priors

delta1 ~ dnorm(0,0.00001) T(0,)

delta2 ~ dnorm(0,0.00001) T(0,)

delta3 ~ dnorm(0,0.00001) T(0,)

# Specify vague proper priors on prevalence of the combined loss of smell/taste symptom

for(i in 1:N) { # [loop over N weeks]

prev.sympt[i] ~ dbeta(1,1)

}

# Likelihood and functional expressions

for(i in 1:N) { # [loop over N weeks]

n.sympt[i] ~ dbin(prev.sympt[i],n.syndrome[i])

# Update P(SARS-CoV-2 | syndrome) [via Bayes theorem] using prevalence of loss of smell/taste,

# and estimated sensitivity & specificity, weighted by prevalence of loss of smell/taste in week

updated.p.sars[i] <- ( (1-prev.sympt[i]) * p.sars.given.syn[i] ) +

( prev.sympt[i] * ( (p.sars.given.syn[i] * sens.sympt) /

((p.sars.given.syn[i] * sens.sympt) + ((1-p.sars.given.syn[i]) * (1-spec.sympt))) ) )

y1[i] ~ dpois(lambda1[i])

lambda1[i] <- delta1 * p.infl.given.syn[i]

y2[i] ~ dpois(lambda2[i])

lambda2[i] <- delta2 * updated.p.sars[i]

y3[i] ~ dpois(lambda3[i])

lambda3[i] <- delta3 * p.other.given.syn[i]

y[i] ~ sum(y1[i],y2[i],y3[i])

}

# Attribution proportions over entire period

x.infl.25 <- sum(y1[1:25]) / sum(y[1:25])

x.sars.25 <- sum(y2[1:25]) / sum(y[1:25])

# Adhoc nodes for attributed % influenza and attributed% SARS-CoV-2 during 13-week influenza season

x.infl.13 <- sum(y1[8:20]) / sum(y[8:20])

x.sars.13 <- sum(y2[8:20]) / sum(y[8:20])

}
